# Risk Factors for Giant Retinal Tears

**Published:** 2010-10

**Authors:** Morteza Mehdizadeh, Mehrdad Afarid, Mohammad Shabanpour Haqiqi

**Affiliations:** Poostchi Ophthalmology Research Center, Department of Ophthalmology, Shiraz University of Medical Sciences, Shiraz, Iran

**Keywords:** Giant Retinal Tear, Risk Factors, Retinal Detachment

## Abstract

**Purpose:**

To evaluate the risk factors associated with giant retinal tears.

**Methods:**

This retrospective study was performed on medical records of 150 patients who had undergone retinal detachment surgery. Age, sex, history of trauma, lens status (phakic, pseudophakic, or aphakic), and high myopia were evaluated in association with giant retinal tears.

**Results:**

Of 150 patients with retinal detachments, 99 subjects (66%) were older than 30 years while 51 (34%) were 30 years of age or younger. Overall, 26 (17.3%) patients had giant retinal tears. Controlling for all variables, only age had a significant correlation with giant retinal tears. Each year of advancing age was associated with a 6% decrease in the incidence of giant retinal tears.

**Conclusion:**

Young age is a significant risk factor for development of giant retinal tears.

## INTRODUCTION

A giant retinal tear is one that extends 90 degrees or more circumferentially.^1^ Giant retinal tears are a challenging form of retinal break because of their complications.

Proliferative vitreoretinopathy (PVR) is one of the late complications of giant retinal breaks and the leading cause of surgical failure.^2^ Increased access to the exposed retinal pigment epithelium (RPE) allows greater spillage of cells and pigment into the vitreous cavity and on the retinal surface, thereby increasing the risk of PVR.^3^ Giant retinal tears in pediatric patients (younger than 16 years) are more likely to be associated with PVR which may be due to a delay in diagnosis or the greater wound healing response in children.^4^

Other mechanisms predisposing to PVR include the presence of vitreous hemorrhage and breakdown of the blood-ocular barrier leading to production of cytokines which could further stimulate cellular proliferation. Choroidal detachment or hypotony may also result from the inflammation following a giant retinal tear.

Giant retinal tears may arise spontaneously, but approximately 25% of cases occur in association with ocular trauma.^5^ The fellow eye of patients who have experienced a spontaneous giant retinal tear is at an increased risk of developing giant retinal tears, retinal detachment, or both.^6^

Previous studies have reported various risk factors for giant retinal tears; these include trauma, high myopia, aphakia and pseudophakia, and young age.^4^–^6^

Since giant retinal tears can adversely affect the prognosis of retinal detachment surgery, we decided to study patients who were operated for retinal detachment at our center and evaluate risk factors predisposing to this type of retinal tear.

## METHODS

This retrospective study was performed on consecutive patients who had undergone retinal detachment surgery at Khalili hospital over a two-year period from March 2007 to March 2009. Medical records of these patients were reviewed and subjects with giant retinal tears were identified.

Data sheets were designed and patient information including age, sex, lens status (phakic, pseudophakic, or aphakic), laterality (right or left eye), final diagnosis (giant retinal tear or no giant retinal tear), and history of trauma were taken into account. The patients were stratified into two age groups: older than 30 years and 30 years of age or younger. High myopia was defined as spherical equivalent refractive error exceeding -6 D.

Statistical analysis was performed to determine risk factors for giant retinal tears. Logistic regression analysis, was performed to control for confounding factors using the forward stepwise (Wald) method. P-values less than 0.05 were considered to be statistically significant.

## RESULTS

Medical records of 150 patients who had undergone retinal detachment surgery during the aforementioned period were evaluated. These included 98 (65.3%) male and 52 (34.7%) female subjects with mean age of 42.7±20.4 and median age of 43 (range, 3 to 87) years. Ninety-nine (66%) patients were older than 30 years, and 51 (34%) were 30 years of age or younger. Retinal detachment involved the right eye in 73 (48.7%) subjects and the left eye in 73 (48.7%) patients. Four patients (2.6%) had bilateral retinal detachment.

Overall, 44 (29.3%) patients had history of significant trauma prior to retinal detachment including 34 (34.7%) male and 10 (19.2%) female subjects (P=0.035). A total of 117 (78%) patients were phakic, 29 (19.3%) were pseudophakic and 4 (2.7%) were aphakic. Thirty-six (24%) cases were highly myopic including 17 (17.3%) male and 19 (36.3%) female patients; high myopia was more prevalent in female patients (P=0.009).

Twenty-six (17.3%) patients including 20 (76.9%) male and 6 (23.1%) female subjects were identified to have giant retinal tears. Of these 7 (26.9%) subjects were older than 30 years whereas 19 (73.1%) were 30 years of age or younger; 15 (57.7%) had history of trauma; 23 (88.5%) eyes were phakic, 2 (7.7%) were pseudophakic, and 1 (3.8%) was aphakic. Six (23.1%) subjects with giant retinal tears had high myopia. The giant retinal tear involved the right eye in 14 patients and the left eye in 11 others; both eyes were involved in one patient.

Logistic regression analysis was performed to control for confounding factors. All variables (age, sex, trauma, myopia, and lens) were entered into the model, but only age had a significant correlation with giant retinal tears. For each year of advancing age, there was a 6% decrease in the incidence of giant retinal tears.

## DISCUSSION

A giant retinal tear is a full thickness retinal break that extends circumferentially for 90 degrees or more in the presence of posterior vitreous detachment.^6^ Risk factors for giant retinal tears according to previous studies include trauma, high myopia, aphakia and pseudophakia, and young age.^4^–^6^ We also evaluated these risk factors and found the most influential to be age.

Giant retinal tears may arise spontaneously, but in some reports approximately 25% of the cases are associated with trauma.^5^ However, the presumed association between trauma and giant retinal tears may be biased. Some patients may have had an overlooked trauma months or weeks before the diagnosis, while others may have exaggerated the severity of any trauma. This bias would affect retrospective studies to a larger extent. In the current study, after controlling for all confounding factors, no correlation was observed between a history of trauma and giant retinal tears.

In the present series, no association was detected between gender and giant retinal tears. However, since trauma may increase the incidence of giant retinal tears, male subjects may have a higher incidence of such tears due to being more prone to trauma.

High myopia is one of the risk factors for retinal tears, especially following trauma.^7^ In highly myopic eyes, even when the contusion is indirect or not severe, it may produce giant retinal tears.^7^ In our study however, no significant association was observed between high myopia and giant retinal tears.

It is well established that the risk of retinal tears and detachment increases after cataract surgery, but after controlling for confounding factors, the current series revealed no statistically significant increase in giant retinal tears in pseudophakic and aphakic eyes.

A systematic review of population-based epidemiologic studies on rhegmatogenous retinal detachment (RRD) has shown that the incidence of RRD demonstrates significant geographic variation ranging from 6.3 to 17.9 cases in 100,000 persons. The overall incidence of RRD is not well established and varies between studies according to ethnicity and is strongly associated with age, high myopia, and certain vitreoretinal degenerations.^8^ In our study, 17.3% of patients who had undergone retinal detachment surgery had giant retinal tears.

Cataract surgery in older patients increases the incidence of retinal tears and detachment. However, giant retinal tears, which are a special form of retinal break, have not yet been demonstrated to be correlated with cataract surgery.

Age is an important factor that can influence the incidence of retinal detachment and giant retinal tears. In the present study, the incidence of giant retinal tears decreased with advancing age. Our data showed that for each year increase in age, the incidence of giant retinal tears decreased by 6 percent. This finding is compatible with other studies which have demonstrated that patients with giant retinal tears tend to be younger.^4^

Based on our results, young age is a significant risk factor predisposing to giant retinal tears. Tight vitreoretinal adhesions in young patients and greater predisposition to ocular trauma could be the main causes for this finding. Although trauma, high myopia, aphakia, and pseudophakia were not identified as independent risk factors, they may predispose to giant retinal tears in young patients. In older subjects these factors may lead to retinal detachment by formation of other types of retinal breaks.

Extensive vitreous liquefaction (EVL) with condensation of the vitreous base has been correlated with giant retinal tears in some studies.^9^ Although giant retinal tears were also found in eyes with uniform posterior vitreous detachment (PVD), they were less extensive and located more posteriorly than eyes with EVL.^9^

The importance of giant retinal tears is that the rate of severe postoperative PVR and unsuccessful retinal reattachment is especially high despite advances in surgical technique.^10^

Some studies support prophylactic treatment for all high-risk fellow eyes of subjects with non-traumatic giant retinal breaks.^11^ Prophylactic treatment may be justified if the risk of complications of treatment is lower than that of retinal detachment.

In summary, we evaluated major risk factors for giant retinal tears such as high myopia, trauma, age, and lens status. Young age was the only significant risk factor associated with giant retinal tears. However other risk factors may have been overlooked. Further studies with a larger sample size should be designed for this purpose. Based on the findings of the current study and previous ones, fellow eyes of patients with non-traumatic giant retinal tears, especially patients younger than 30 years of age, may be considered at high risk and therefore prophylactic treatment may be warranted in such cases.
